# Synthesis and Evaluation of Rutin–Hydroxypropyl β-Cyclodextrin Inclusion Complexes Embedded in Xanthan Gum-Based (HPMC-*g*-AMPS) Hydrogels for Oral Controlled Drug Delivery

**DOI:** 10.3390/antiox12030552

**Published:** 2023-02-22

**Authors:** Abid Naeem, Chengqun Yu, Zhenzhong Zang, Weifeng Zhu, Xuezhen Deng, Yongmei Guan

**Affiliations:** 1 Key Laboratory of Modern Preparation of Traditional Chinese Medicines, Ministry of Education, Jiangxi University of Chinese Medicine, Nanchang 330004, China; 2 School of Pharmacy, Jiangxi University of Chinese Medicine, Nanchang 330004, China

**Keywords:** hydrogels, flavonoids, anti-oxidants, inclusion complexes, oral formulations

## Abstract

Oxidants play a significant role in causing oxidative stress in the body, which contributes to the development of diseases. Rutin—a powerful antioxidant—may be useful in the prevention and treatment of various diseases by scavenging oxidants and reducing oxidative stress. However, low solubility and oral bioavailability have restricted its use. Due to the hydrophobic nature of rutin, it cannot be easily loaded inside hydrogels. Therefore, first rutin inclusion complexes (*RIC*) with hydroxypropyl-β-cyclodextrin (HP-βCD) were prepared to improve its solubility, followed by incorporation into xanthan gum-based (hydroxypropyl methylcellulose-grafted-2-acrylamido -2-methyl-1-propane sulfonic acid) hydrogels for controlled drug release in order to improve the bioavailability. Rutin inclusion complexes and hydrogels were validated by FTIR, XRD, SEM, TGA, and DSC. The highest swelling ratio and drug release occurred at pH 1.2 (28% swelling ratio and 70% drug release) versus pH 7.4 (22% swelling ratio, 65% drug release) after 48 h. Hydrogels showed high porosity (94%) and biodegradation (9% in 1 week in phosphate buffer saline). Moreover, in vitro antioxidative and antibacterial studies (*Staphylococcus aureus*, *Pseudomonas aeruginosa*, and *Escherichia coli*) confirmed the antioxidative and antibacterial potential of the developed hydrogels.

## 1. Introduction

Flavonoids consist of a wide variety of naturally occurring compounds. The variable phenolic structures found in plants include flavonols, anthocyanidins, flavan-3-ols, flavones, flavanones, and isoflavones. These substances are found in vegetables, stems, fruits, grains, roots, barks, and flowers and have received considerable scientific and therapeutic attention [1]. Since ancient times, flavonoids have played a fundamental role in several treatments. Several flavonoids can scavenge free radicals, enhance antioxidant functions, possess anti-inflammatory properties, exhibit anti-tumor properties, and display anti-thrombogenic properties and antibacterial properties [2].

Rutin (RT), a flavonoid, also called vitamin P, can be found in a wide variety of foods, including apples (350 to 4780 μg/g), citrus fruits (2.7 to 8106.7 µg/g), and tea (303 to 479 μg/g) [3]. Rutin is an important metabolite of *Ruta graveolens* [4]. It exhibits strong antioxidant properties (which are comparable to those of ascorbic acid and butylated hydroxytoluene), anti-inflammatory, antiviral, anticancer, antidiabetic, asthma-reducing, antibacterial, cardiovascular, cholesterol-lowering, and neuroprotective properties [5]. As a result of its polyhydroxy structure, rutin is susceptible to various environmental factors, including temperature (above 75 °C) and pH (in high acidic and alkaline environments). Moreover, rutin is poorly soluble due to the presence of the benzene ring and the hydroxyl group in the same molecule, thereby limiting its use and applications [6]. The use of rutin can be improved by resolving the problem of its solubility. The bioavailability and absorption of drugs are significantly affected by solubility and gastrointestinal permeability [7]. The antioxidant rutin is a quercetin glycoside that cannot be absorbed in its natural form due to its structure [8]. Rutin is primarily hydrolyzed by the caecal microflora in the large intestine [9]. Pure rutin has a bioavailability of approximately 20% when taken orally [10]. Consequently, rutin is not very bioavailable due to its poor solubility in aqueous media (0.8 mg/mL) [11]. The development of novel drug delivery methods will ensure the bioavailability of this promising natural molecule, thereby enabling it to be used to treat chronic human diseases.

Even though rutin is widely used in the pharmaceutical and nutraceutical industries, there have only been a limited number of studies that have explored how to increase its dissolution rate and, ultimately, its bioavailability. This can be accomplished by preparing coprecipitated microparticles or inclusion complexes that contain a hydrophilic polymer [12]. Inclusion complexes can be formed by utilizing cyclodextrins (CDs), cyclic oligosaccharides commonly used for stabilizing and protecting several active compounds, masking their unpleasant odor and taste, and increasing dissolution rates in pharmaceutical and nutraceutical products. The structure of a CD is truncated and resembles a cone [13]. Their hydrophilic external surfaces make them fully water soluble, whereas their hydrophobic interior cavities allow them to accommodate molecules of different sizes, shapes, and degrees of hydrophobicity [14]. Guest/host inclusion complexes are formed primarily through non-covalent interactions, such as hydrophobic and hydrogen bonds and van der Waals forces [15]. Compared to other CDs, α-cyclodextrin does not have a sufficient cavity to encapsulate most active molecules, while γ-cyclodextrin is not economically feasible due to the high cost. Thus, β-cyclodextrin (β-CD) has become the most widely used one [16,17]. 2-Hydroxypropyl-β-cyclodextrin (HP-βCD) represents a chemically modified version of β-cyclodextrin exhibiting enhanced safety properties compared with its naturally occurring parent compound [18]. HP-βCD, a water-soluble oligosaccharide that has been listed as generally recognized as safe (GRAS) by the FDA, has shown promising results in enhancing flavonoids’ solubility [19,20]. In a study conducted by Miyake et al., it was demonstrated that rutin was more soluble and bioavailable when complexed with HP-βCD [21].

Many limitations are associated with traditional drug dosage forms, including dose and delivery limitations arising from fluctuations in drug plasma levels beyond and below the therapeutic range. As a result, this effect will differ according to the biological half-life of the drug, frequency of administration, as well as the rate at which it is released. In addition, chronic diseases require a multi-dose regimen in which patients are unable to adhere to dosing frequencies. As a result of fluctuating plasma drug concentrations and poor patient compliance, conventional dosage forms are being replaced by controlled release dosage forms [22]. Controlled release drug delivery systems (CDDS) offer many advantages, including the capability of maintaining a steady concentration of the drug at the target site, improving patient compliance by reducing dosages and dosage frequency, reducing side effects, and delivering drugs effectively, etc. [23].

Controlled drug release may be achieved by using polymers with known release profiles. In this manner, drug release rate fluctuations are minimized and effectively delivered [24]. Generally, controlled drug release systems are designed to deliver drugs to a targeted area at a predetermined rate for a specified period of time in order to maintain the desired concentration and improve therapeutic efficacy [25]. Hydrogels consist of a crosslinked network of hydrophilic polymers capable of absorbing water solutions without being solubilized [26]. Hydrogels have been used extensively in a variety of fields, including agriculture, food, biotechnology, and pharmacy. Hydrogels are capable of absorbing and releasing substantial amounts of drugs as a result of their swelling properties. Hydrogel swelling behavior directly affects the release rate of the drug [27].

Additionally, hydrogels have several limitations in addition to these advantages. For example, many hydrogels have low mechanical strength, which prevents them from loading drugs, leading to early dissolution or egress from the targeted area. Gels can only be loaded with hydrophilic drugs, whereas hydrophobic drugs may pose problems, such as inadequate amounts of drug loading and decreased homogeneity [28]. Another factor to consider is the rapid and early release of drugs by some hydrogels. Hydrogels are characterized by large pores and high-water contents, which may lead to burst releases of their contents [29]. The advent of natural polymers, particularly polysaccharides, has influenced researchers in recent years to design dosage forms that are biodegradable, biocompatible, renewable, and non-toxic [30]. Xanthan gum is secreted by *Xanthomonas campestris*, and it is an anionic polymer because it contains both glucuronic and pyruvic acid groups. It has attracted considerable attention in the past few years due to its versatility, particularly in critical environments with acidic conditions, high salt levels, and high shear stresses [31]. Additionally, it can conjugate itself to other polymers, peptides, proteins, and nonpeptides, resulting in conjugates that can withstand enzyme degradation and are biocompatible and easily soluble. Since it has a high affinity for water, xanthan gum enhances the solubility of hydrophobic drugs and carriers. In vitro studies have shown that small quantities of xanthan gum can also decrease the rate at which drugs are released [32].

Hydroxypropyl methylcellulose or HPMC, belongs to the hydrophilic class of polymers that can be used to make oral drug delivery systems. It is characterized by a significant degree of swelling and surface activity. Drug release from a hydrogel is affected by its swelling properties. When HPMC polymer chains come into contact with water, they relax and expand, causing drugs to diffuse from the hydrogel [33]. In addition, polymer adsorption to drug surfaces strongly depends on surface activity. It has been shown that the surface activity of hydrophobic drugs is significantly influenced by the adsorption of cellulose ethers containing hydroxypropyl groups. Polymers such as HPMC are often used in solid drug dispersions as rate-controlling agents [34,35].

2-acrylamido-2-methylpropanesulfonic acid (AMPS), a hydrophilic ionic monomer, plays an essential role in preparing hydrogels for drug delivery. Due to the presence of ionizable sulfonic groups in AMPS-based hydrogels, they exhibit a pH-independent swelling behavior. All of these sulfonic groups are completely dissociable irrespective of pH. Moreover, increasing the concentration of AMPS increases the swelling index of hydrogels because of the increase in the number of ionic groups in the hydrogels [36]. Ethylene glycol dimethacrylate (EGDMA) was utilized to crosslink the hydrogels.

Based on the above considerations, the objective of this study was to prepare a hydrogel based on polysaccharides, namely xanthan gum (XG) and hydroxypropyl methylcellulose (HPMC), loaded with hydrophobic drug (rutin) inclusion complexes with 2-hydroxypropyl-β-cyclodextrin (RIC) for oral drug delivery and to control the release of rutin for a prolonged period of time in order to improve its bioavailability. A variety of techniques were used to characterize hydrogel’s structure, including Fourier transform infrared spectroscopy (FTIR), X-ray diffraction (XRD), differential scanning calorimetry (DSC), thermogravimetric analysis (TGA), porosity of the gel network, sol–gel fraction analysis, volume fraction of polymer (V2,s), crosslinking molecular weight (Mc), and biodegradation. Hydrogel swelling and release behavior were also examined using different polymer and crosslinker concentrations in different pH media (pH 1.2 and pH 7.4). Additionally, rutin inclusion complex-loaded hydrogels were evaluated for their antioxidant activities (DPPH and ABTS assays) as well as their antibacterial (*Staphylococcus aureus*, *Pseudomonas aeruginosa*, and *Escherichia coli)* properties.

## 2. Materials and Methods

### 2.1. Materials

Hydroxypropyl methylcellulose (HPMC; MW 470.5 g/moL) was obtained from Rhawn chemical technology, Shanghai, China. Xanthan gum (Viscosity = mPa·s ≥ 1200, MW: 1016.8 g/moL) was obtained from cool chemical science technology, Shanghai, China. Rutin (≥98%; MW: MW: 610.5 g/moL) and sodium bisulfite (SHS) were procured from Shanghai Aladdin biochemical technology, Shanghai, China. Ammonium persulfate (APS), 2-acrylamido -2-methyl-1-propanesulfonic acid (AMPS; MW: 207.25 g/moL), and ethylene glycol dimethacrylate (EGDMA; MW: 198.22 g/moL) was obtained from Sigma-Aldrich St. Louis, MO, USA. Hydroxypropyl beta cyclodextrin (HP-βCD; MW:1541.5 g/moL), DPPH (2,2- diphenyl-1-picryhydrazyl), ABTS (2,2’-azino-bis (3-ethylbenzothiazoline-6-sulfonic acid) and Cefepime HCL were obtained from Meilune biological company (Dalian,China).

Bacterial strains such as *Staphylococcus aureus* (*S. aureus*: ATCC25923HBJZ005), *Pseudomonas aeruginosa* (*P. aeruginosa*: ATCC27853HBJZ017), and *Escherichia coli* (*E. coli*: ATCC25922HBJZ087) were obtained from Qingdao Hope Biotechnology, Co, Ltd., Qingdao, China.

### 2.2. Preparation of Rutin Inclusion Complexes with HP-βCD (RIC)

The inclusion complexes were prepared by following the method of Vijaya Sri et al. with slight modification [37]. HP-βCD was dissolved in deionized water, while rutin was dissolved in methanol and protected from light to prevent its degradation. The molar ratio used to prepare inclusion complexes was 1:1 for HP-βCD and rutin. The samples were mixed and placed on a magnetic stirrer for 72 h at a speed of 350 rpm/min. After thoroughly mixing, the supernatant was removed by centrifugation and placed in a refrigerator at −20 °C overnight. Finally, rutin inclusion complexes (*RIC*) were obtained after freeze-drying the samples for 2 days. The entrapment efficiency (EE%) of rutin in *RIC* was 72.30%, drug loading (DL%) was 21.99%, and the yield was 89.07%.

### 2.3. Synthesis of Xanthan Gum-Based (HPMC-g-AMPS) Hydrogels

**Figure 1:**
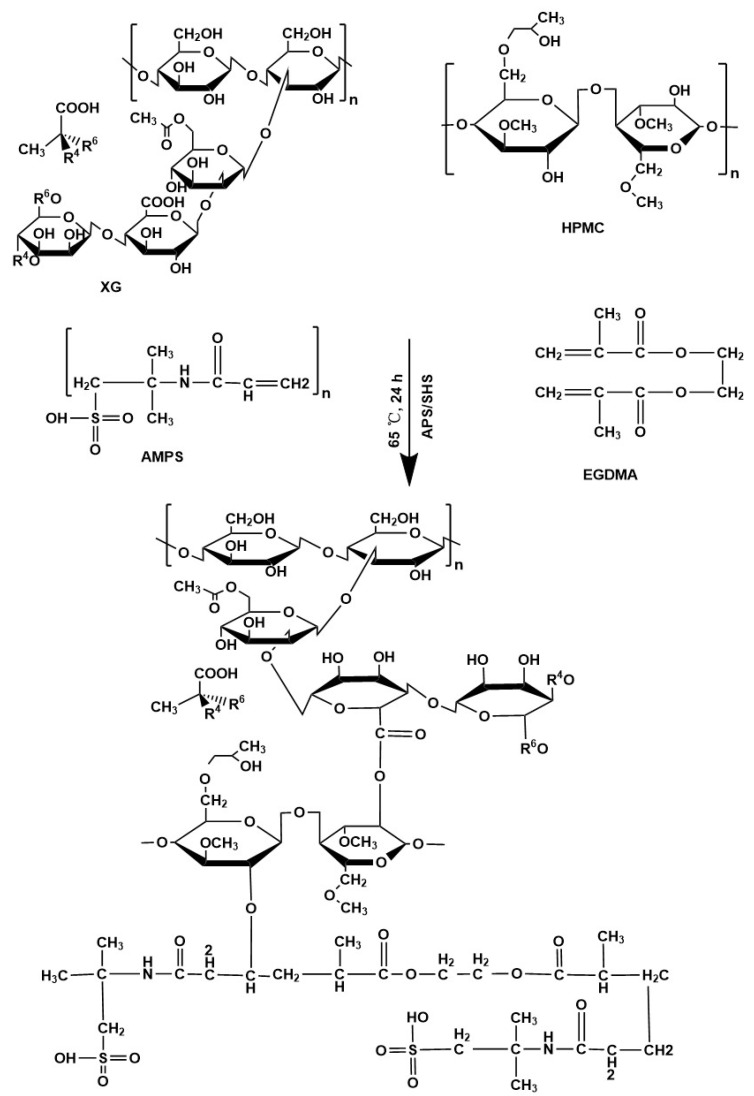
Proposed chemical structure of the xanthan gum-based (HPMC-*g*-AMPS) hydrogels.

Different batches of hydrogels were synthesized using the free radical polymerization method with slight modification by grafting monomers onto polymer networks [38]. HPMC, xanthan gum, APS/SHS, AMPS, and EGDMA were carefully weighed and placed in labelled glass bottles. The appropriate amount of water was added to all the labelled vials. Xanthan gum was dissolved without lumps or precipitations by stirring continuously at 40 °C, while HPMC was dissolved without any precipitation at room temperature. We used APS and SHS as initiators and co-initiators for the reaction, respectively. The SHS was dissolved in distilled water, and the APS was added slowly to prepare the initiator mixture. The clear aqueous solution of AMPS was also prepared by stirring continuously at room temperature. While stirring continuously, a clear solution was obtained by incorporating the initiator/co-initiator solution drop-by-drop into the monomer solution. HPMC solution was stirred continuously while the AMPS and initiator/co-initiator mixture was poured drop-by-drop into it. This solution was slowly added to the xanthan gum solution and well blended. The EGDMA was poured slowly into the mixture and stirred well to ensure even mixing. Finally, a sufficient amount of water was added to the reaction mixture and thoroughly mixed. After this, the mixture was put in an ultrasonic bath while nitrogen bubbles were used to eliminate any remaining air. The clarified solution was then covered by aluminium foil. Samples were then placed in a preheated water bath for 1 h at 50 °C, followed by an overnight increase to 65 °C. After 24 h, clear, transparent hydrogels were formed. Once the hydrogel had been formed in the water bath, it was cooled in the glass molds to room temperature. It was then removed from the molds and cut into 8 mm diameter discs. The discs were transferred to individually labeled Petri dishes after washing with ethanol and water (50:50). One week after being stored at 40 °C, the weight of the hydrogels became constant. Table 1 shows a series of xanthan gum-based (HPMC-*g*-AMPS) hydrogel compositions with varying amounts of polymer, monomer, and crosslinker. Figure 1 shows a schematic representation of xanthan gum-based (HPMC-*g*-AMPS) hydrogels [33,39,40,41].

#### RIC Loading in Xanthan Gum-Based (HPMC-*g*-AMPS) Hydrogels

Rutin was used as a model drug, and its inclusion complexes were formed with HP-βCD and then loaded into hydrogels by the swelling-diffusion technique. Briefly, *RIC* was dissolved in 0.2 M phosphate buffer of pH 7.4. Then, dried and preweighed hydrogel discs were added to *RIC* solution for 48–72 h. The hydrogel discs were removed and dried to a constant weight. Drug loading in hydrogels was determined using the following formula. Moreover, drug loading was also verified by extracting all the drugs from the hydrogels.

(1) Drug loading=Drug loaded hydrogel−Unloaded hydrogel

### 2.4. Characterization

#### 2.4.1. ^1^H NMR and Fourier Transform Infrared Spectroscopy (FTIR)

^1^H NMR spectra were measured utilizing TMS as an internal standard and D_2_O as a solvent on a Bruker AV 500 MHz (Bruker BioSpin, Zurich, Switzerland). TOPSPIN software was used to process the spectra. Attenuated total reflectance (ATR) spectroscopy was employed to analyze drug-formulation interactions using a Spectrum Two FTIR spectrometer (Perkin Elmer, Buckinghamshire, UK). Spectra of *RIC*-loaded and unloaded hydrogel samples and purified components were acquired between the scanning ranges of 400 and 4000 cm^−1^.

#### 2.4.2. Thermal Analysis (TGA and DSC)

The thermal stability of the sample was examined by the Exstar TG/DTA6300TG thermogravimetric analyzer (SII Nano, Tokyo, Japan) and differential scanning calorimetry (Perkin Elmer, Buckinghamshire, UK) [42]. A thermogravimetric analyzer was used to measure temperature-dependent weight change. Initially, reference standards were used to calibrate the weight profile. AMPS, XG, HPMC, *RIC*, rutin, and HP-βCD were placed in aluminium pans (0.5 to 5 mg each). The inert nitrogen flow rate of 10 mL/min was employed to measure weight loss with a 10 °C/min scanner. The melting points of AMPS, XG, HPMC, *RIC*, rutin, HP-βCD, and the prepared hydrogels were analyzed by differential scanning calorimetry (DSC). A sapphire standard was used to calibrate calorimeters for their heat capacity. A standard of indium was used to determine the cell constant and temperature.

#### 2.4.3. X-ray Diffraction (XRD)

X-ray diffraction (TD-3500 X-ray diffractometer, China) was used to measure crystallinity by irradiating the target (CuKα) at 30 kV and 20 mA [43]. The scans were performed at a speed of 2 degrees per minute at a slope of 2θ between 10° and 60°. Software such as Jade/MDI was utilized to analyze the data. Crystallinity can be determined by examining the peaks of the substances examined using XRD. The crystallinity of materials can be determined by sharp peaks, while amorphous properties can be determined by diffuse peaks. Measurements were performed on AMPS, HPMC, XG, *RIC*, rutin, HP-CD, and *RIC*-loaded and unloaded preparations.

#### 2.4.4. Morphological Analysis

The surface morphology of fabricated hydrogels was investigated by scanning electron microscopy (Quanta 250, FEI company, Eindhoven, The Netherlands). Dry hydrogels were cut to the appropriate size and adhered to aluminium tubing with double-sided tape [44]. Gold was sputtered on the stubs in a high-vacuum evaporator under an argon atmosphere. Photomicrographs of the coated samples under 15 kV accelerated current were obtained to determine the surface morphology.

#### 2.4.5. Mechanical Properties Analysis

Tensile strength (TS) and elongation at break (EAB) were measured using a TA.XT plus texture analyzer (Stable Micro Systems, Godalming, UK) equipped with a stainless steel spherical probe with a diameter of 5 mm and a testing speed of 1.0 mm/s. TS and EAB are determined by measuring the force and displacement exerted by the probe upon breaking the hydrogel (swollen form) [45].

(2) $$\mathrm{TS}=\frac{\mathrm{Fm}}{\mathrm{Th}}$$

(3) $$\mathrm{EAB}=\frac{\sqrt{D^{2}+R^{2}}}{R}-1$$

In this equation, Th is the hydrogel thickness, and Fm is the maximal force exerted by the probe. Distance D between the probe first making contact with the hydrogel and the point at which it breaks the hydrogel is the radius of the orifice plate (R).

#### 2.4.6. Sol–Gel Study

The Soxhlet apparatus was used to examine the Sol–gel fraction of the formulated hydrogel by extracting it in deionized boiling water for 13 h [33]. Hydrogel slabs measuring 4 mm in thickness were tested in a Soxhlet apparatus. The slabs were removed from the apparatus after 13 h of extraction and dried at 40 °C until they reached a constant weight. The following equations were used to measure the sol and gel fractions.

(4) $$\mathrm{Sol}\;\mathrm{fraction}\;\%=\frac{E1\;–\;E2}{E2\;}\times 100$$

(5) Gel fraction=100−Sol fraction

whereas E1 represents the weight of the hydrogel at the beginning (initial weight), and E2 represents the final weight (extracted).

#### 2.4.7. Porosity Study

The porosity of each hydrogel formulation was analyzed and evaluated using a solvent replacement approach. Hydrogel discs (Q1) of a known amount of weight were submerged in absolute ethanol (purity > 99.9%) for 5 days. After 5 days, the hydrogel discs were collected, wiped with filter paper to remove any remaining solvent, and then weighed again (Q2). The diameter and thickness of the discs were measured. Porosity was calculated using the provided equation [46].

(6) $$\mathrm{Porosity}\;\mathrm{percentage}\;\left(\%\right)=\frac{Q2\;–\;Q1\;}{\rho v\;}\times 100$$

whereas ρ is the ethanol density, and v represents the hydrogel volume in swelled form.

#### 2.4.8. Biodegradation Study

Xanthan gum-based (HPMC-*g*-AMPS) hydrogels were tested for biodegradation at a pH of 7.4 and a temperature of 37 ± 0.5 °C. Dry hydrogel discs were weighed and soaked in a pH 7.4 buffer solution for various durations (1, 2, 3, 4, 5, 6, and 7 days). Hydrogel discs were removed at the appropriate time, dried in a vacuum oven set to 40 °C, weighed again, and then placed in a buffer solution at a pH of 7.4. This procedure was repeated until further hydrogel disc degradation was detected [47]. The following formula can be used to calculate hydrogel degradation:

(7) $$D=\frac{p1-p2}{p1}$$

whereas D represents the degradation process; P1 represents the initial, dry sample weight; and P2 represents the final, immersed sample weight at time (t).

### 2.5. Characteristics of the Synthesized Hydrogel Polymer Network

The most important indicators of the structure and properties of a hydrogel are the swelling-state polymer volume fraction (V2,s), crosslinking molecular weight (Mc), solvent interaction parameters (χ), and the number of linkages between pairs of crosslinks (N) [38,48,49].

#### 2.5.1. Diffusion Coefficient

The degree of diffusion of a substance is determined by the nature of the polymer and its segmental mobility. The diffusion coefficient was estimated using the following formula:

(8) $$\;D=\pi {\left(\frac{h.\theta }{4.\mathrm{qeq}}\right)}^{2}$$

whereas qeq represents hydrogel swelling, ɵ is the slope of swelling curves, and h represents the hydrogel disc thickness prior to swelling.

#### 2.5.2. Polymer Volume Fraction (V2,s)

V2,s represents the fraction of polymer in a fully swollen state. Volume swelling (Veq) data were collected for two pH values (1.2 and 7.4) to estimate polymer volume fraction. Polymer volume fraction was determined using the following formula:

(9) $$V2,s=\frac{1}{\mathrm{Veq}\;}$$

#### 2.5.3. Average Molecular Weight between Crosslinks (Mc)

Mc can be used to estimate the degree of crosslinking of polymer networks. The following equation can be used to determine it.

(10) $$\mathrm{Mc}\;=\frac{\mathrm{dpVs}\left({V\;}^{\frac{1}{3}}2,s-V2,\frac{s}{2}\right)}{\ln\left(1-V2,s\right)+V2,s+{\chi V}^{2}2,s}$$

whereas dp refers to the polymer density. Vs reflects the solvent’s molar volume and χ provides information on the interaction of the polymer with the solvent according to Flory–Huggins theory.

#### 2.5.4. Solvent Interaction Parameters (χ)

It can be determined by the following equation.

(11) $$\chi =\frac{\ln\left(1-V2,s\right)+V2,s}{V^{2}2,s\;}$$

whereas V2,s represents swollen gel volume fraction.

#### 2.5.5. Crosslinking Units (N)

The data of Mc was utilized to determine N. It was calculated using the following equation.

(12) $$N=\frac{2\mathrm{Mc}}{\;\mathrm{Mr}\;}$$

Mr refers to the repeating unit’s molar mass. The following equation can be used for its calculation:

(13) $$\mathrm{Mr}=\frac{\mathrm{mHPMCMHPMC}\;+\;\mathrm{mXGMXG}\;+\;\mathrm{mAMPSMAMPS}+\mathrm{mEGDMAMEGDMA}}{\mathrm{mHPMC}+\mathrm{mXG}+\mathrm{mAMPS}+\mathrm{mEGDMA}}$$

whereas m is the mass and M represents the molar mass of HPMC, XG, AMPS, and EGDMA, respectively.

### 2.6. Hydrogels Swelling Study

The swelling behavior of fabricated hydrogels was studied at different pH values [50]. Hydrogel disc weights were initially recorded in dry form, and then the discs were placed in simulated gastric fluid (pH 1.2) and intestinal fluid (pH 7.4) at 37 °C. Disc weights were recorded periodically at defined time intervals until constant weights were obtained. The following formula was used for calculating the swelling ratio.

(14) $$\;\mathrm{ESR}=\frac{\mathrm{Ws}-\mathrm{Wd}}{\mathrm{Wd}}\times 100$$

whereas Ws represents the mass of a swollen hydrogel at a set time interval, and Wd represents the weight of a dried hydrogel.

### 2.7. In Vitro Release Study and Kinetics Data Modeling

The USP dissolution apparatus II was used for in vitro rutin release testing to determine drug release patterns based on pH [51]. Dissolution mediums consisted of simulated gastric and intestinal fluid with pH 1.2 and 7.4, respectively. Drug-loaded hydrogel discs were weighed (approximately 1 g) before being inserted into both mediums (900 mL). In each dissolution basket, the medium volume was maintained at 900 mL. The experiment was carried out at a temperature of 37 °C. The paddle speed was adjusted to 50 revolutions per minute. Samples were obtained from the buckets at predefined intervals, diluted with fresh buffer solution, and examined with a UV–Vis spectrophotometer (T6 New Century; Beijing GM) at a wavelength of 359 nm (the calibration curve of rutin was prepared in the abovementioned buffer and used for the determination of the drug release from the hydrogels).

(15) $$\mathrm{Drug}\;\mathrm{release}\%=\frac{\left(\mathrm{Amount}\;\mathrm{of}\;\mathrm{released}\;\mathrm{drug}\right)}{\left(\mathrm{Amount}\;\mathrm{of}\;\mathrm{loaded}\;\mathrm{drug}\;\right)}\times 100$$

Drug release from hydrogels can be influenced by various factors, including the relaxation of polymer chains, the hydrogel’s swellability, the drug’s nature, and the release medium’s pH. The swelling of hydrogels is primarily the result of solvent diffusion required for this process during controlled release. Several models were used to determine the drug release pattern, including zero order, first order, Higuchi, and Korsmeyer–Peppas models. The given equations were used.

(16)  Zero−order kinetics Ft=K0t

whereas the apparent rate constant is denoted by K0, and the amount of drug released at time t is denoted by Ft.

(17) $$\;\mathrm{First}\;\mathrm{order}\;\mathrm{kinetics}\;\ln\left(1-F\right)=-K1t$$

whereas F denotes the quantity of drug released during time t and k1 denotes the first-order release rate constant.

(18) $$\;\mathrm{Higuchi}\;\mathrm{model}\;F=K2t^{\frac{1}{2}}$$

whereas F is the quantity of drug released at time t, while K2 is the Higuchi constant. The model is defined by the following hypotheses: (I) the solubility of the drug is lower than its initial concentration in the framework, and (II) there is one-directional diffusion of the drug.

(19) $$\;\mathrm{Korsmeyer}-\mathrm{Peppas}\;\mathrm{model}\;\frac{\mathrm{Mt}}{M¥}=K3t^{n}$$

whereas Mt represents the water mass obtained at time t, while M¥ represents the water mass that was obtained at equilibrium. K3 is a constant that considers the gels’ geometrical and structural features, and n is the release exponent. When the value of n is 0.45, it indicates a Fickian release mechanism; however, if the value of n is greater than 0.45 but lower than 1, it demonstrates a non-Fickian release mechanism.

### 2.8. Antioxidant Studies

#### 2.8.1. DPPH Antioxidant Activity

DPPH radical scavenging analysis was used to evaluate the antioxidant properties of the synthesized hydrogels. Predetermined quantities of samples were immersed in methanol for 24 h at room temperature in the dark. The sample solution was then combined with 1 mL of DPPH methanol solution (0.1 mM). The mixture was then thoroughly agitated and incubated for 30 min in a dark environment. The DPPH scavenging activity was then estimated by measuring the absorbance of the solution with a UV–Vis spectrophotometer at 517 nm. It was calculated using the following equation [52].

(20) $$\mathrm{DPPH}\left(\%\right)=\frac{A0-A}{A0}\times 100$$

whereas A0 and A represent the absorbance of the reference and test samples, respectively.

#### 2.8.2. ABTS Antioxidant Activity

The ability of RIC-loaded hydrogels to scavenge free radicals was measured using the ABTS assay. The mixture of 7.4 mM ABTS and 2.4 mM potassium persulfate was combined in a 1:1 ratio and left to sit at room temperature for an entire night in order to induce radicalization of the ABTS molecules. Each hydrogel was incubated for 30 min at 37 °C with ABTS solution. The absorbance of the sample was measured at a wavelength of 730 nm. ABTS’s radical-scavenging efficiency was determined using the following formula [53].

(21) $$\mathrm{ABTS}\;\mathrm{scavenging}\;\mathrm{effect}\;\left(\%\right)=\frac{A0-A1}{A0}\times 100\;$$

whereas A0 stands for ABTS absorbance and A1 for sample absorbance.

### 2.9. Antibacterial Study

Hydrogels were tested for their antibacterial properties using a disk-diffusion method with Gram-negative and Gram-positive organisms in a nutrient agar medium. The agar medium was sterilized at 121 °C after it was prepared. A sterile broth was used to grow the bacterial strain. *Staphylococcus aureus*, *Pseudomonas aeruginosa*, and *Escherichia coli* were cultivated on agar medium under aseptic conditions and then transferred into Petri dishes for solidification. We divided the plates into four sections: a blank hydrogel, *RIC*-loaded hydrogels, a positive control (Cefepime at 1 mg/mL), and a negative control. These plates were incubated for 24 h in an incubator. The zone of inhibition was determined for each sample in order to compare the results [54].

(22) $$\mathrm{Percentage}\;\mathrm{inhibition}=\frac{\mathrm{Zone}\;\mathrm{of}\;\mathrm{inhibition}\;\mathrm{of}\;\mathrm{test}\;\mathrm{sample}\;\left(\mathrm{mm}\right)}{\mathrm{Zone}\;\mathrm{of}\;\mathrm{inhibition}\;\mathrm{of}\;\mathrm{standard}\;\mathrm{drug}\;\left(\mathrm{mm}\right)}\times 100$$

### 2.10. Statistical Analysis

All the data were expressed as mean ± SD. The statistical differentiation between pairs of data was determined using a two-way ANOVA and Tukey’s post hoc test. To determine the significance of the difference between swelling and drug release profile, *p*-values were determined and were denoted as * *p* < 0.05, ** *p* < 0.01, and *** *p* < 0.001.

## 3. Results and Discussion

### 3.1. ^1^H NMR and FTIR Analysis

**Figure 2:**
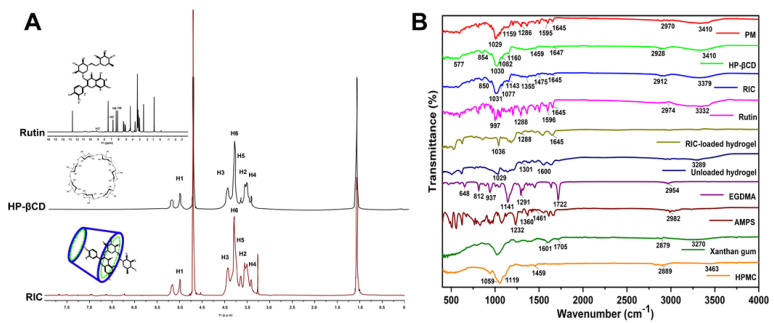
^1^H NMR of the rutin inclusion complexes (**A**) and FTIR analysis of the pure components and developed hydrogels (**B**).

The ^1^H NMR technique provides direct confirmation of the inclusion of the guest into the HP-βCD cavity [55]. The inclusion of rutin in HP-βCD cavities is reflected by changes in protons’ electronic and chemical environments, resulting from the complexation process and reflected in changes in chemical shift (∆δ) measurements. This chemical shift provides evidence as to which part of the guest molecule is inserted into the CD cavity. The ^1^H NMR spectra of HP-βCD, rutin, and their inclusion complex are shown in Figure 2A. The ^1^H chemical shifts of free HP-βCD and rutin were consistent with previous studies and are shown in Table 2 [6]. Consequently, the inclusion complexation of rutin with HP-βCD has a negligible effect on the values of its H-2 protons (0.002). However, H-5 (narrow side) and H-6 protons exhibit significant chemical shift changes, 0.015 and −0.021 ppm respectively. Moreover, rutin addition shielded H-4 and H-6 on the inner surface of HP-βCD while leaving H-1, H-3, and H-5 unshielded when added to the HP-βCD. The positive sign of the Δδ ppm indicates a downfield displacement, whereas a negative sign indicates an upfield displacement (Δδ = δ_complex_ − δ_free_) [56]. These results indicate that rutin forms inclusion complexes with HP-βCD.

FTIR spectra of HPMC, xanthan gum, AMPS, rutin, EGDMA, *RIC*, HP-βCD, unloaded hydrogel, and the drug-loaded hydrogel are shown in Figure 2B. HPMC exhibited a distinct broadband region at 3463 cm^−1^ corresponding to O-H stretching vibrations. Bands at 2890 and 1060 cm^−1^ were assigned to C-H and C-O bonds [57]. Additionally, HPMC showed an important stretching vibration at 1459,1119 cm^−1^ assigned to CH_3_ and C-O-C bonds [58]. Xanthan gum exhibits stretching vibrations at 3270 cm^−1^ due to axial deformation of the O-H. At 2879 cm^−1^, the peak results from the stretching vibrations of the C-H group, while at 1705 cm^−1^, the peak results from stretching vibrations of the C-O group, while the bands near 1601 cm^−1^ are due to the axial deformation of the C-O portion of the enol [59]. FTIR spectra of AMPS revealed a peak at 1461 cm^−1^ corresponding to the binding vibration of CH_2_. AMPS spectrum shows vibrational peaks at 1360 cm^−1^ (-C-O stretching), 2982 cm^−1^ (-CH stretching of -CH_2_), and 1230 cm^−1^ (symmetrical S=O stretching), which are all characteristic of AMPS. EGDMA spectrum revealed peaks at 1713 cm^−1^ associated with C=O stretching vibrations, whereas the peaks at 1633, 1291, and 1153 cm^−1^ are attributed to C=C and C-O stretching vibrations in the symmetric and asymmetric esters, respectively [60]. Absorption bands were seen for rutin at 3332 cm^−1^ (OH stretching), 2974 cm^−1,^ and 997 cm^−1^ (C-H vibration), and 1300 to 1000 cm^−1^ (rutin C-O stretching vibration) [61]. The peaks at 1645 cm^−1^ belong to the C=O stretching vibration, and at 1596 cm^−1^ to the C=C stretching vibration of the aromatic structure of rutin [62]. HP-βCD showed peaks at 3410 cm^−1^ due to stretching vibrations of OH and at 2928 cm^−1^ due to C-H vibrations. The characteristic bands at 1160 cm^−1^ and 1030 cm^−1^ were attributed to C-O stretching vibrations. The results are consistent with those obtained by other researchers [63]. In the *RIC* FTIR spectrum, the broad band at 3379 cm^−1^ indicates the existence of a –OH group in the structure, whereas the peak at 1131 cm^−1^ corresponds to the C-O stretching vibration in HP-βCD. Rutin’s C=O structure is represented by the peak at 1645 cm^−1^, and the presence of multiple peak bands indicates that rutin and HP-βCD mixed thoroughly to form the inclusion complex. The FTIR spectrum of blank hydrogel showed an absorption band at 3289 cm^−1^ (OH stretching), and C-O bonding around 1600 cm^−1^. The peak data indicate that these substances are crosslinked successfully. FTIR spectra of *RIC*-loaded hydrogels displayed a different spectrum than those of their parent components. The bands appearing at 1645 cm^−1^ were considered as the peaks of the C=O group. The peak at 1036 cm^−1^ belongs to C-O stretching vibration. The emergence of new peaks, and the presence of functional groups, suggests that the polymers used were successfully crosslinked.

### 3.2. TGA Analysis

**Figure 3:**
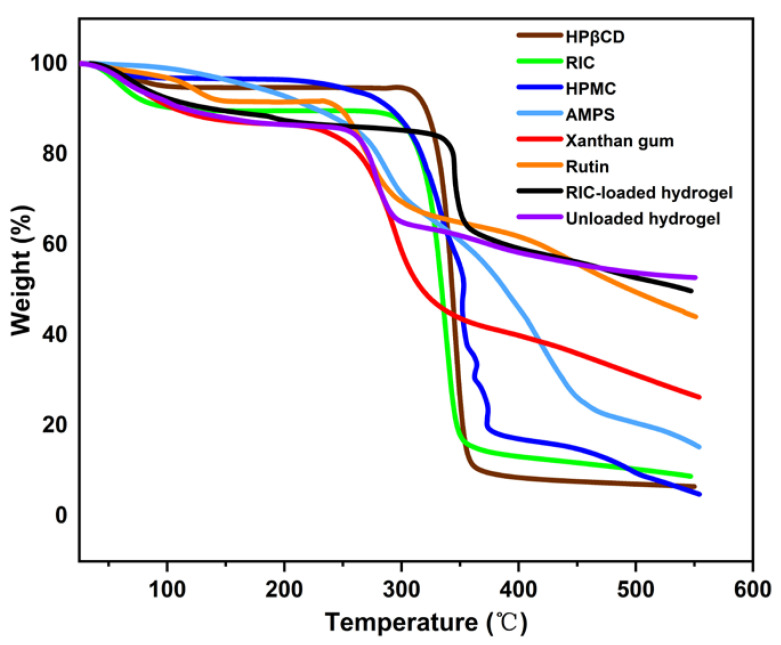
TGA analysis of HP-βCD, rutin, HPMC, XG, AMPS, *RIC*, unloaded and *RIC*-loaded hydrogels.

TGA was conducted to assess the thermal stability of the polymers, inclusion complexes, and synthesized hydrogels (Figure 3). According to the TGA analysis of AMPS, there was a 6% reduction in weight at 208 °C, followed by a 20% reduction between 210 and 250 °C, indicating water and moisture loss. During the decomposition of the sulfonic acid group, a weight loss of 20% was detected within the temperature range of 250 °C to 340 °C [64]. HPMC polymers lose approximately 4% of their water at temperatures below 100 °C; however, it is thermally stable to a temperature of more than 300 °C [65]. Xanthan gum loses weight by 12% from 25 °C to 180 °C, possibly due to moisture absorption from the sample’s surface and the bulk, but the weight loss rate decreases after 296 °C [66]. In the case of the HP-βCD samples, the weight loss below 80 °C was attributed to water evaporation, whereas the weight loss between 305 °C and 380 °C could be attributed to HP-βCD degradation, which is similar to that reported by other researchers [63]. The weight loss observed in the rutin sample between 30 °C and 134 °C is attributed to the evaporation of water, and the peak of weight loss between 248 °C and 364 °C may be attributed to rutin weight loss. *RIC* exhibited a change in temperature from 50 °C to 80 °C due to water loss and a change from 280 °C to 348 °C as a result of *RIC* degradation. Initially, hydrogel samples lose weight from 50 °C to 260 °C due to water dissipation and polymer bonding breakdown. When the temperature is raised to 260 °C and 280 °C, 20% of the weight is lost. Thus, it shows that the formulation has higher thermal stability than pure reactants.

### 3.3. Differential Scanning Calorimetry (DSC)

**Figure 4:**
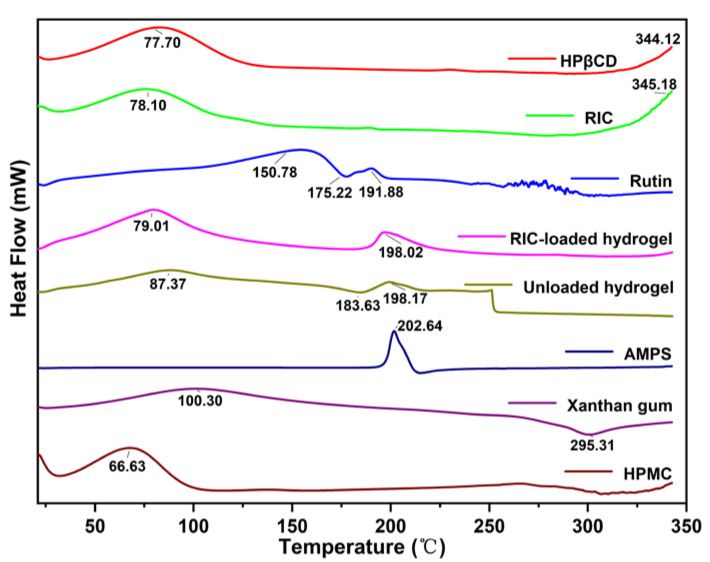
DSC analysis of rutin, HP-βCD, XG, HPMC, *RIC*, AMPS, unloaded and *RIC*-loaded hydrogels.

The HPMC exhibits an endothermic peak of free water at 50–100 °C, which suggests that some water molecules are bonded to the HPMC. Several small peaks indicate a relatively disordered crystalline state [67]. A strong absorption peak can be observed for xanthan gum near 100 °C, consistent with Umme Hani et al.’s experimental results [68]. The glass transition’s temperature was estimated to be close to 290 °C. AMPS indicated a sharp endothermic peak around 202 °C, which is attributed to the decomposition of sulphonic acid groups [69]. DSC data of the rutin sample showed an endothermic phase between 56 °C and 150.78 °C resulting from water loss. As in previous studies, mass loss from 175.22 °C to 191.88 °C was attributed to rutin melting [61]. Based on the DSC data for HP-βCD, the melting peak of HP-βCD is located around 344.12 °C, while the endothermic peak corresponding to water molecules occurs at 77.70 °C. This change may be triggered by hydrogen bonds between water’s H group and the OH group of HP-βCD [63]. Hydrogels have an exothermic peak located around 198.17 °C, showing the presence of AMPS in the formulation. The newly developed structure has resulted in a thermally stable formulation (Figure 4).

### 3.4. XRD Analysis

**Figure 5:**
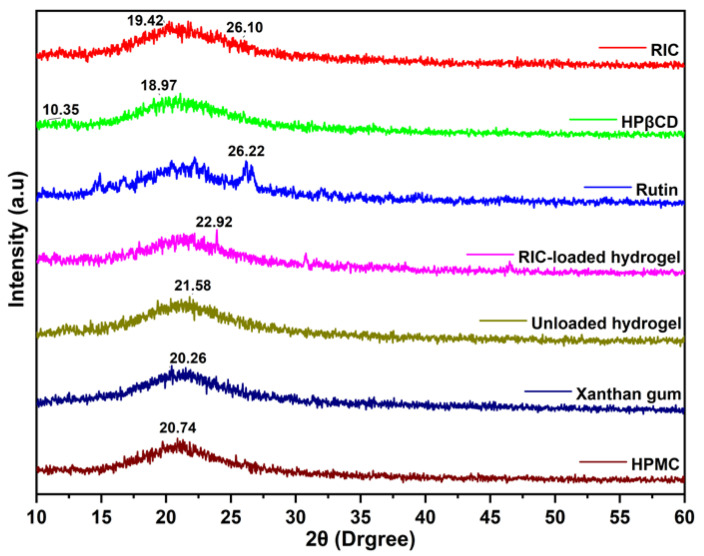
XRD pattern of formulation components, inclusion complexes, and synthesized hydrogels.

Figure 5 shows the XRD spectra of all the polymers and fabricated hydrogels. HPMC shows a diffraction peak at 2θ = 20.74° [70]. The amorphous nature of xanthan gum is evidenced by the absence of clear, sharp peaks and the presence of a broad, amorphous peak in the vicinity of 2 theta of 20°. Other researchers have reported similar results regarding xanthan gum’s amorphous nature [71]. HP-βCD displays two peaks at 2θ = 10.35° and 18.97°, which indicate that HP-βCD has amorphous characteristics. Other researchers have also obtained similar results. A sharp diffraction peak is observed for rutin at 2θ = 26.22°, which is typical for rutin with high crystalline properties. The diffractogram of the *RIC* sample reveals a broad peak at 2θ = 19.42°, while the unloaded hydrogel sample shows a broad peak at 2θ = 21.58°. However, the diffractogram of drug-loaded hydrogels demonstrates only one broad peak at 2θ = 22.92°, while no other intense drug peaks were displayed in their respective regions. The cause of this may be the physical interaction of the drug with the polymeric blend, which interfered with its purity and consequently diminished its crystal lattice properties.

### 3.5. SEM Analysis

**Figure 6:**
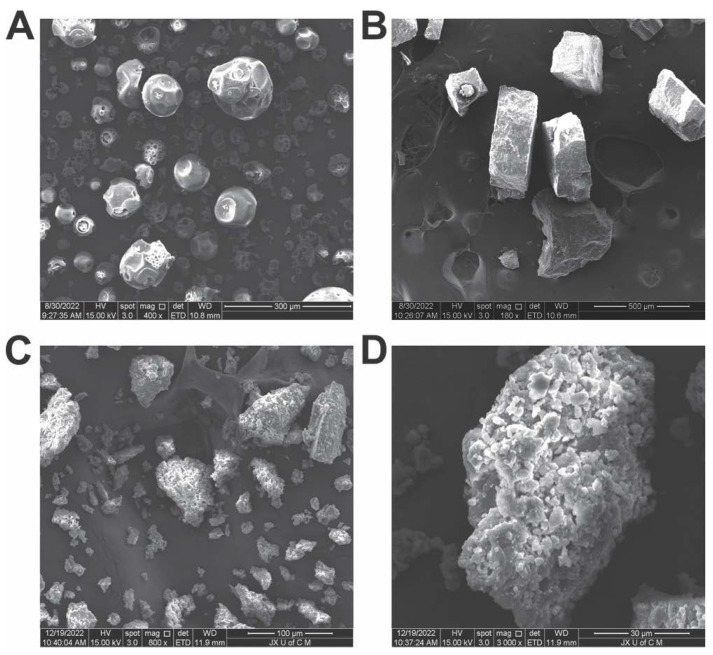
SEM images of HP-βCD (**A**), *RIC* (**B**), hydrogels at 200× (**C**), and hydrogels at 400× (**D**).

SEM images of HP-βCD show a porous, spherical cavity-filled ball-like structure [72]. However, upon complex synthesis with rutin, the porous and spherical morphology of HP-βCD was replaced by a regular folded structure [73]. These results indicate that after inclusion, a different solid phase with a different morphology was observed, which was consistent with the results obtained from the XRD analysis. Hydrogel microphotographs show highly dense, wavy, irregular surfaces (Figure 6). The polymeric gel network may collapse fractionally during the drying procedure, resulting in a rough surface. Furthermore, hydrogels were found to have a high density of crosslinked polymeric networks, which had pores and channels that were effective for entrapping drugs [74]. Porous structures cause swelling and the release of drugs due to the media being received at their surfaces. Micropores gradually absorb fluid as macropores fill up, followed by macropores filling up. It has been demonstrated that a smooth surface and a solid mass play a significant role in the stability of a polymeric network, as shown by the hydrogel system.

### 3.6. Mechanical Properties Analysis

Hydrogels should be evaluated for their mechanical properties, including their tensile strength (TS) and elongation at break (EAB), before being used for drug delivery. Tensile strength was increased by the increase in EGDMA content (Table 3) [75]. In addition, the tensile strength of the composite increases gradually as the HPMC content increases. The mechanical strength of the gel will decrease when the AMPS content increases, likely due to increased electrostatic repulsion and osmotic pressure. Xanthan gum is also a biopolymer that increases the tensile stress and cohesion of bonds, resulting in stronger bonds and an increase in tensile strength [76]. The hydrogels showed better mechanical properties than the previously reported hydrogels [48,77].

### 3.7. Sol–Gel Analysis

The sol–gel fraction was assessed for all hydrogel compositions. Hydrogels are divided into two fractions: the sol fraction, which is composed of the uncrosslinked portion, and the gel fraction, which is composed of the crosslinked portion [32]. The sol fraction refers to the small amount of uncrosslinked hydrogel that remains after the polymerization reaction since there are no reactive sites. Each hydrogel formulation was subjected to sol–gel analysis to determine crosslinking and uncrosslinked percentages. This technique primarily quantifies the number of uncrosslinked polymers [78]. The hydrophilic nature of AMPS causes more chemical reactions to occur as the concentration of AMPS increases, resulting in a thicker gel. HPMC is a type of polymer that increases gel fraction with increased content. EGDMA acts as a crosslinking agent that induces gel formation due to the crosslinking process [79]. The gel fraction will increase correspondingly when EGDMA content increases [80].

### 3.8. Porosity Study

**Figure 7:**
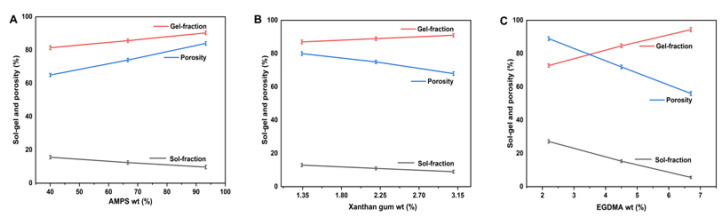
Effect of AMPS (**A**), EGDMA (**B**), and xanthan gum (**C**) on sol–gel and porosity of the fabricated hydrogels [82,83].

Porosity plays an instrumental role in hydrogel swelling, drug loading, and drug release. As pore sizes increase, swelling increases, and as a result, drug loading and release increase. A higher concentration of HPMC increases the porosity of fabricated hydrogels. As the reaction mixture becomes more viscous, bubbles are prevented from escaping, resulting in increased porosity. These factors lead to the development of interconnected channels and increasing porosity. Porosity decreases due to tight junctions and crosslinking of bulk densities as EGDMA concentration increases. The porosity of the gel can also be affected by xanthan gum. HXA-9 exhibits a smaller porosity than other groups due to an increased amount of xanthan gum [81]. Furthermore, HPMC also affects the porosity of the gel. Porosity increases with increasing amounts of HPMC, and similar results have been reported by other researchers. Due to the increased concentration of EGDMA, porosity decreased due to the formation of tight junctions and increased crosslinking. A higher concentration of AMPS will result in greater porosity since the sulfonate groups will generate more electrostatic forces. AMPS contains a hydrophobic alkyl group, which can reduce hydrogen bond interactions by forming hydrophobic microregions. This leads to an increase in pore size and network size in the hydrogel formulation (Figure 7), which is per other studies [33].

### 3.9. Biodegradation Analysis

**Figure 8:**
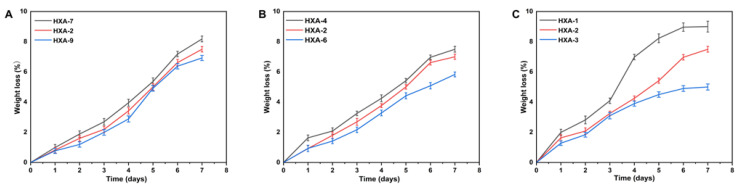
Effect of different formulation components on the biodegradation of hydrogels, such as (**A**) xanthan gum (HXA-2,7,9), (**B**) AMPS (HXA-2,4,6), and (**C**) EGDMA (HXA-1,2,3).

Figure 8A–C illustrates the results of the biodegradation experiment performed to determine the degradation rate of the prepared hydrogel at different time periods. Degradation speed is affected by weight ratios, and with increasing EGDMA, hydrogel degradation speed has been found to be slow. This may be due to the generation of functional groups, which produce large quantities of free radicals. Consequently, these free radicals are capable of strengthening the polymerization reaction, resulting in a slower degradation rate as they contribute to the strength of the crosslinked compound’s hydrogel. Xanthan gum decreases gel degradation as the polymer content increases, which is consistent with the results of other researchers [84].

### 3.10. Structural Parameters of Hydrogels

In this study, various structural parameters of prepared hydrogels were examined, including the average molecular weight of crosslinks Mc (degree of polymer crosslinking), polymer volume fraction V2,s (amount of solvent taken up and held within the network), intersolvent interaction parameter χ, a repeating unit between crosslinks N, and diffusion coefficient D [80]. Table 4 lists the different structural parameters of fabricated hydrogels. The hydrogel’s maximum absorption and holding capacity depend on these characteristics; hence, calculating them is crucial. V2, S, and χ rose with the increased concentration of EGDMA, indicating the development of tighter and stiffer gel formations. The values of Mc decreased, and N increased as EGDMA concentration increased because higher amounts of EGDMA are responsible for forming stronger crosslinks.

### 3.11. Swelling Behavior

**Figure 9:**
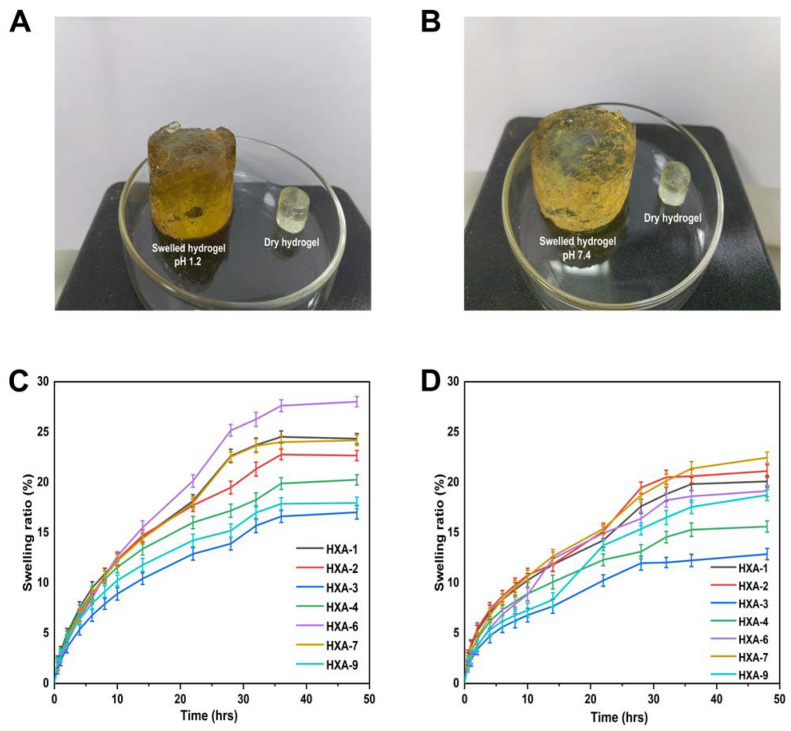
Physicochemical properties of hydrogels when swollen at different pH levels, such as (**A**) pH 1.2 and (**B**) pH 7.4. Hydrogel swelling curves at pH 1.2 (**C**) and pH 7.4 (**D**).

Various concentrations of polymer (HPMC, xanthan gum, AMPS, and EGDMA) were used to prepare hydrogels to investigate the effects on swelling ratio in different media. Whenever hydrogel composites are soaked in different media, their bonds break off due to their hydrophilic nature and crosslinking of all polymers. Figure 9 shows the swelling rate of the hydrogel at various pH values over time. The results showed that the hydrogel swelling was higher at pH = 1.2 (28% swelling ratio) compared to pH = 7.4 (22% swelling ratio). This increased swelling is due to the ionization of hydroxyl (-OH) functional groups in the hydrogel matrix [54].

Hydrogel swelling behavior is determined by functional groups that may be ionized or protonated, interactions between hydrophilic and hydrophobic groups, and chain relaxation. The developed hydrogels exhibited high swelling kinetics at pH 1.2 and low swelling at pH 7.4. The behavior may result from the protonation of functional groups within AMPS [85]. The swelling degree of hydrogels increased with the increase in AMPS concentration, possibly as a consequence of AMPS containing a large number of—CONH_2_ and—SO_3_OH groups, which combine with water molecules after ionization, increasing the swelling of hydrogels, as was previously observed by other researchers [48].

The hydrophilicity of XG and the presence of o-acetyl and pyruvyl residues in XG may contribute to its ability to increase the swelling degree of the hydrogel. In addition, swelling will also increase with an increase in the ratio and concentration of XG. Other studies have also reported similar findings [85]. EGDMA acts as a crosslinking agent, so as the concentration of EGDMA increases, the crosslinking density of hydrogels will increase, which will reduce the porosity of the hydrogel and reduce the amount of water penetrating the mesh, leading to reduced swelling and vice versa [86].

### 3.12. Drug Release Behaviour and Kinetics Modelling

Drug release in the buffer of pH 1.2 ranged from 40.12% to 75.17%. The drug release rate from HXA-6 was the highest at pH = 1.2 (75.17%), whereas the drug release rate from HXA-3 was the lowest at pH = 1.2 (40.12%). Drug release rates in the buffer with pH = 7.4 were 39.77%~70.25%. The highest drug release was observed with HXA-7 (70.25%), and the lowest was HXA-3 (33.77%). The release curve for *RIC* indicated that its release differed with pH buffers, with the maximum release occurring in pH buffers of 1.2. The maximum drug release rate was 75.17% after 48 h.

**Figure 10:**
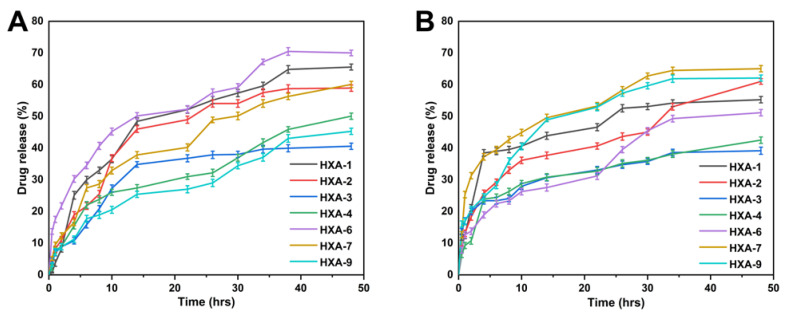
The drug release curve of hydrogel under pH conditions of 1.2 (**A**) and 7.4 (**B**).

Hydrogel discs are immersed in buffer/water, and as a result of the osmotic pressure gradient, water molecules diffuse into the polymer network. Diffusion of water causes the hydrogel discs to swell, which causes channels to open, causing the drug to be released (Figure 10). A regression coefficient value near 1 was used to determine which model best fits the release data. The regression coefficients (r) for samples (HXA-2, HXA-7, HXA-9) containing varying concentrations of xanthan gum, samples with varying concentrations of the crosslinker EGDMA (HXA-1, HXA-2, HXA-3) and samples containing varying concentrations of AMPS (HXA-2, HXA-4, HXA-6) followed the Korsmeyer–Peppas model and indicated that the drug release mechanism was diffusion-based. The release exponent (n) values of all drug-loaded samples (HXA-1, HXA-2, HXA-3, HXA-4, HXA-6, HXA-7, HXA-9) following the Korsmeyer–Peppas model were less than 0.5, which indicated Fickian diffusion (Table 5) [87].

### 3.13. Antioxidation Analysis

**Figure 11:**
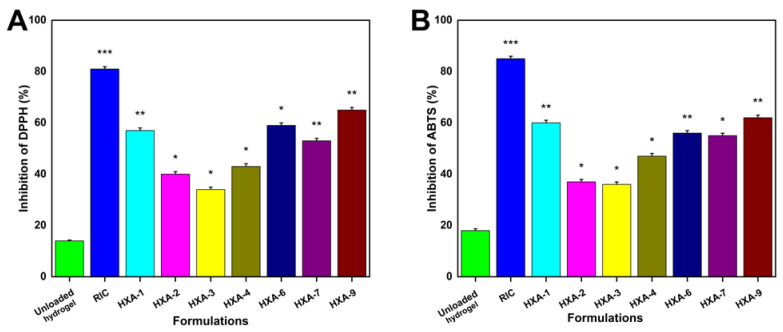
Hydrogel antioxidant activity was assessed using the DPPH (**A**) and the ABTS assay (**B**). (Here, * shows the *p* value < 0.05, ** *p* < 0.01, and *** *p* < 0.001).

DPPH and ABTS scavenging abilities of hydrogels were evaluated by measuring the scavenging efficiency, as depicted in Figure 11A,B. Four formulations (HXA-1, HXA-6, HXA-7, and HXA-9) exhibited superior antioxidant activity than other formulations. These formulations all showed higher swelling and release levels and higher feeding concentrations of xanthan gum. Rutin has excellent antioxidant properties, as well as the ability to inhibit cancerous growth, mutations, and the growth of bacteria [88]. The antioxidant properties of rutin have also been demonstrated in vitro and in vivo. Rutin’s antioxidant properties were demonstrated in a previous study by its ability to scavenge ROS and reduce oxidative stress [89]. Rutin shows significant antioxidant properties when loaded into a hydrogel. Xanthan gum (XG) is produced by the anaerobic fermentation of a species of bacteria named Xanomonas brassica. There are several unique properties of XG, including its non-toxic nature, biodegradability, intrinsic ability to function as an immunity agent, antioxidant property, and stability. It has drawn much attention as a good scavenger of reactive oxygen species due to its mixture of hydroxyl, reducible sugar, pyruvate, and o-acetylation components. The XG formulation can effectively reduce intracellular ROS levels and alleviate the effects of oxidative stress [90].

### 3.14. Antibacterial Study

**Figure 12:**
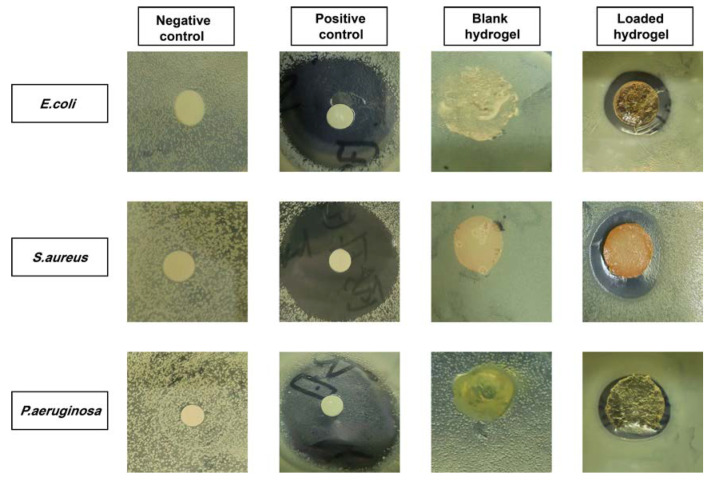
Different inhibition zones were observed against *E. coli*, *S. aureus*, and *P. aeruginosa* using the hydrogels.

This study examined the antibacterial activity of the formulation against Gram-positive and Gram-negative bacteria, and their inhibition zones are illustrated in Figure 12. The results demonstrated that no zones were observed in the negative control and blank hydrogel groups, while clear zones were observed in the positive control, i.e., 27, 29, and 23 mm, and in the *RIC*-loaded hydrogels, i.e., 13, 15, and 8 mm against *E. coli*, *S. aureus*, and *P. aeruginosa,* respectively. According to the bacteriostatic formula, the inhibitory percentages of the *RIC*-loaded hydrogels for *E. coli*, *S. aureus*, and *P. aeruginosa* were 48.14%, 51.72%, and 34.78%, respectively. Broad-spectrum antibiotic cefepime kills both Gram-positive and Gram-negative bacteria [88]. Observations showed that cefepime had a smaller antibacterial activity against Gram-negative bacteria than Gram-positive bacteria. This is possible due to the structure of their cell walls. Gram-negative bacteria possess a thin cell wall composed of three layers: an inner membrane, a peptidoglycan cell wall, and an outer membrane. Gram-positive bacteria have a thick cell wall. However, they do not have an outer membrane as Gram-negative bacteria do. The outer membrane of Gram-negative bacteria serves as a protective barrier from the environment. Hence, Gram-positive bacteria demonstrate better antibacterial activity or a larger inhibition zone than Gram-negative bacteria.

## 4. Conclusions

In this study, the rutin inclusion complexes with HP-βCD (*RIC*) were developed first in order to improve the aqueous solubility of the drug in aqueous media. The entrapment efficiency (EE%) of rutin in RIC was 72.30%, the drug loading (DL%) was 21.99%, and the yield was 89.07%, indicating successful inclusion complexation. Then, xanthan gum-based (HPMC-*g*-AMPS) controlled release hydrogel was fabricated using a free radical polymerization technique, incorporating natural (xanthan gum) and semi-synthetic polymers (HPMC) as well as grafting monomers (AMPS). FTIR, XRD, TGA, and DSC measurements confirmed the formation of HP-βCD-rutin inclusion complexes (*RIC*), the hydrogel network, and the successful loading of the drug (*rutin*), and SEM analysis showed that the hydrogels are porous. The hydrogels showed a slightly higher swelling at pH 1.2 (28% swelling) compared to 22% swelling at pH 7.4 after 48 h. Moreover, a slightly higher drug release was observed in the synthesized hydrogels after 48 h at pH 1.2 (70%) than at pH 7.4 (65%), with Fickian diffusion becoming the dominant mechanism. Increased polymer ratios and monomer concentrations achieved longer drug release times and improved mechanical properties. Moreover, the hydrogels were found to be highly porous (94% porosity) and biodegradable (9% weight loss in 1 week). In addition, the developed hydrogels demonstrated significant antioxidant activity in the DPPH assay (inhibition of 65%) and the ABTS assay (inhibition of 62%). In addition, the hydrogels demonstrated excellent antibacterial properties against Gram-positive bacteria such as *E. coli (zone* of inhibition of 13 mm) and *S. aureus* (zone of inhibition of 15 mm), as well as Gram-negative bacteria *P. aeruginosa* (zone of inhibition of 8 mm). In summary, HP-βCD inclusion complex-loaded xanthan gum-based (HPMC-*g*-AMPS) hydrogels could offer a viable alternative for the prolonged delivery of hydrophobic (which is difficult to load inside hydrogels) as well as hydrophilic drugs.

## Figures and Tables

**Table 1:** The composition of fabricated xanthan gum-based (HPMC-*g*-AMPS) hydrogels.

Formulation	HPMC (g)	Xanthan Gum (g)	APS/SHS (g)	AMPS (g)	EGDMA (g)
HXA-1	0.5	0.5	0.3/0.3	20	**0.5**
HXA-2	0.5	0.5	0.3/0.3	20	**1**
HXA-3	0.5	0.5	0.3/0.3	20	**1.5**
HXA-4	0.5	0.5	0.3/0.3	**12**	1
HXA-5	0.5	0.5	0.3/0.3	**20**	1
HXA-6	0.5	0.5	0.3/0.3	**28**	1
HXA-7	0.5	**0.3**	0.3/0.3	20	1
HXA-8	0.5	**0.5**	0.3/0.3	20	1
HXA-9	0.5	**0.7**	0.3/0.3	20	1

Note: Bold values indicate higher feeding amounts.

**Table 2:** Changes in chemical shift (∆δ, ppm) of HP-βCD in free and complexed state.

Protons	Δ(free)	Δ(complex)	Δδ
HP-βCD			
H1	4.982	4.996	0.014
H2	3.550	3.552	0.002
H3	3.933	3.946	0.013
H4	3.509	3.496	−0.013
H5	3.643	3.658	0.015
H6	3.793	3.772	−0.021

**Table 3:** Mechanical characteristics and drug (*RIC*) loading of hydrogels.

F. Codes	Thickness (mm)	TS (N/mm)	EAB (%)	*RIC* Loaded per 1 g Hydrogel (g)
HXA-1	1.425	0.356	31.3	0.365
HXA-2	1.334	0.668	59.1	0.451
HXA-3	1.298	1.117	79.9	0.235
HXA-4	1.255	0.774	66.7	0.249
HXA-5	1.334	0.668	59.1	0.451
HXA-6	1.415	0.651	60.1	0.334
HXA-7	1.554	0.548	58.6	0.482
HXA-8	1.334	0.668	59.1	0.451
HXA-9	1.610	0.801	67.4	0.322

**Table 4:** Flory–Huggins network parameters of xanthan gum-based (HPMC-*g*-AMPS) hydrogels (data are presented as mean ± standard deviation, n = 3).

F. Codes	V_2,s_	χ	M_c_	M_r_	N	D × 10^−5^ (cm^2^ s^−1^)
HXA-1	0.041 ± 0.003	0.514 ± 0.041	4406.1 ± 1.509	231.99 ± 0.002	37.98 ± 0.98	0.309 ± 0.061
HXA-2	0.044 ± 0.005	0.515 ± 0.018	3369.4 ± 2.067	231.22 ± 0.001	29.14 ± 1.02	0.219 ± 0.023
HXA-3	0.058 ± 0.006	0.520 ± 0.005	2357.5 ± 0.765	230.48 ± 0.002	20.45 ± 0.97	0.011 ± 0.001
HXA-4	0.049 ± 0.004	0.516 ± 0.031	3157.9 ± 1.251	244.91 ± 0.003	25.83 ± 1.07	0.041 ± 0.003
HXA-5	0.044 ± 0.005	0.515 ± 0.018	2298.9 ± 1.367	231.22 ± 0.001	29.14 ± 0.69	0.219 ± 0.023
HXA-6	0.035 ± 0.008	0.511 ± 0.016	3577.4 ± 2.004	224.82 ± 0.007	31.82 ± 1.16	0.028 ± 0.007
HXA-7	0.041 ± 0.006	0.514 ± 0.011	4563.2 ± 1.669	224.01 ± 0.002	40.74 ± 1.21	0.672 ± 0.052
HXA-8	0.041 ± 0.003	0.514 ± 0.041	4406.1 ± 1.509	231.99 ± 0.002	37.98 ± 0.98	0.309 ± 0.061
HXA-9	0.044 ± 0.005	0.515 ± 0.018	3369.4 ± 2.067	231.22 ± 0.001	29.14 ± 1.02	0.219 ± 0.023

**Table 5:** The release kinetics of rutin from xanthan gum-based (HPMC-*g*-AMPS) hydrogels crosslinked with EGDMA at different pH levels.

F. Codes	pH	Zero Order		First Order		Higuchi Model		Korsmeyer–Peppas Model	
		K_o_ (h^−1^)	r^2^	K_1_ (h^−1^)	r^2^	K_2_ (h^−1^)	r^2^	r^2^	n
HXA-1	1.2	0.913	0.9492	0.011	0.9666	5.227	0.9951	0.9954	0.453
	7.4	0.647	0.9450	0.008	0.9566	3.725	0.9907	0.9910	0.413
HXA-2	1.2	1.001	0.9386	0.013	0.9616	5.755	0.9923	0.9932	0.455
	7.4	0.772	0.9456	0.009	0.9597	4.445	0.9914	0.9917	0.412
HXA-3	1.2	0.793	0.9446	0.010	0.9605	4.562	0.9957	0.9964	0.427
	7.4	0.619	0.9317	0.007	0.9446	3.612	0.9905	0.9931	0.361
HXA-4	1.2	0.626	0.9274	0.007	0.9416	3.628	0.9915	0.9952	0.401
	7.4	0.541	0.9236	0.006	0.9358	3.156	0.9886	0.9934	0.364
HXA-5	1.2	1.001	0.9386	0.013	0.9616	5.755	0.9923	0.9932	0.455
	7.4	0.772	0.9456	0.009	0.9597	4.445	0.9914	0.9917	0.412
HXA-6	1.2	1.217	0.9644	0.017	0.9846	6.851	0.9946	0.9952	0.536
	7.4	0.876	0.9007	0.011	0.9120	5.048	0.9406	0.9405	0.417
HXA-7	1.2	0.970	0.9512	0.012	0.9699	5.553	0.9955	0.9958	0.471
	7.4	0.786	0.9383	0.010	0.9546	4.512	0.9861	0.9865	0.441
HXA-8	1.2	1.001	0.9386	0.013	0.9616	5.755	0.9923	0.9932	0.455
	7.4	0.772	0.9456	0.009	0.9597	4.445	0.9914	0.9917	0.412
HXA-9	1.2	0.607	0.9287	0.007	0.9422	3.521	0.9917	0.9953	0.395
	7.4	0.519	0.8716	0.006	0.8830	3.017	0.9258	0.9274	0.378

## Data Availability

Not applicable.
